# Dentists and Dentistry as Seen by Our Patients

**Published:** 1892-08

**Authors:** W. H. Wright

**Affiliations:** Brandon, Vt.


### Dentists and Dentistry as Seen by Our Patients.

READ BY DR. W. H. WRIGHT, BRANDON, VT.

Read before the Vermont State Dental Society, March, 1892.

This world contains a great deal of trouble; but a convention of dentists represents more misery to the square inch than any other assemblage of a like number of men, and the memory of past experiences with them brings back recollections of days that lasted only too long.

Of all the pain that flesh is heir to, what can equal the tooth-ache? From infancy to old age it is one constant struggle with teeth, first to grow them, then to keep them, and lastly to be rid of them altogether, then to invest in false ones.

Just why the First Great Cause did not make the jaw-bone and teeth all one—permanent—and without nerves, has always been a mystery; but after the mistake had been made, dentists had to be evolved, and it appears they have come to stay.

It is astonishing what an amount of ignorance an educated man may possess; good common sense is not found alone in books, it is a simple homely quality, which is not always inherited, and the bluest blood may lack its vigorous strength.

No profession needs more good sense than a dentist's. The recipient of the miserable experiences of people needs discernment and a silent tongue. No doubt there are many cranks among patients, but what will reduce a strong healthy person to idiocy sooner than a jumping tooth-ache?

It would be well, if when the dental student had received his diploma, he should be required to undergo, at least, one trial of inhaling ether, the attendant pleasure of having teeth extracted, with a piece of jawbone attached, his most sensitive tooth bored out and filled, with all the minor punches and scrapes which are thrown in, how many dentists would there be in the country? not enough for a corporal's guard.

Did anybody ever hear of a dentist having a tooth pulled? *Never.* Did anybody ever know of a dentist wearing false teeth?

The *oldest inhabitant* never heard of a case. Did any dentist ever have the nerve to have a tooth killed? *No*, with a big *N*. Dental students talk glibly of nerves, a most delicate and sensitive thing, to be tenderly cared for and approached with fear and trembling, that a tooth pressing on a nerve will cause all the "ills flesh is heir to," but as soon as their diploma is framed, they proceed to eradicate every vestige of pity from their system, invest in a dental chair manufactured of iron, made strong on purpose to hold aches (which they encourage for business purposes), then they solicit victims; and they scrape, and file, and drill, never losing an opportunity to touch the sorest spot, they will beguile you with their silvery tongue, but the first good chance, *ough*, and you wish you had died in infancy, and you *know* the top of your head has lifted a foot at least. After your very best feelings have been *harrowed* and *dragged*, they will chuck in the filling, take your money, smile blandly upon you, and invite you to call again. If there were more good mechanics among dentists, there would be more well-fitting false teeth, it makes one feel pleasant to have them tilt from one side to the other while trying to eat, and very likely to leave a corn. Dentists talk fluently about molars and grinders, incisors and eye-teeth, that every tooth has its own motion, spring and nerve, and those nerves all lead somewhere (nerves usually let folks know where they are), but many a man who could build a rail fence scientifically has been swallowed up in the dental profession, who ought not to have anything more *nervy* than a rail to operate on.

It is not expected that a dentist will be all saint-like, as it is seldom he has saints to deal with, neither does he cut his wisdom teeth earlier than his patients, but he should aspire to be *the best* in the profession, take pride in doing his *work well*, and not trust to his professional dignity, or the apprentice in the back room to do a *good job*.

In these days of microbes, bacteria, germs, etc., is it a wonder that any one is left *alive*, and dentists are to be held responsible for the health of future generations. Who can tell just what spasm the digestive organs undergo, when the teeth do not act with equal pressure while chewing a tough steak?

The medical fraternity have always occupied the "high seats," but a thorough mastication of food is the best doctor, and we look to the dentist for a preventive from the all-destroying germs of disease. *The surest sign of health is a clean mouth*, and with all the facilities for a scientific education, laboratories, lecture rooms, and improved mechanical appliances, it would seem as if our troubles were nearly over.

But we ought not to fail in noticing the other side of our subject. Every cloud has a silver lining, and the dental operator is no exception. As we approach his realm it looks like a cloudy darkness, and everything in it wears the forbidding aspect of a chamber of horrors; but when all is over, the pain gone, and our teeth made as good as new, we leave with light and not ungrateful hearts. It is then that we recognize the substantial benefits bestowed by the dental professors upon our generation in relieving an evil which they did not cause, and in saving us from the fate of being a toothless race, to be fed on pap the rest of our days. Thousands of happy patients can testify to the relief which they have found through the skill acquired by much labor and study, and are ever ready to honor those who have conferred upon them exemption from suffering, and so added both to the power and the enjoyment of their lives. It was well we did not need such services, but needing we should be poor without them. It is one of the many benefits conferred by science upon us that those who are acquainted with its lore find there is a practical power to bless the world.

Let us have good men in the profession, *with all* the words imply, men who make a *thorough study* of their *business* and are *never too old to learn* newer methods. Men of solid worth, GENTLEMEN. The community look up to professional men for an example. They are expected to have, added to their natural talents, the refining influences of our institutions of learning, and are expected to do their *level best*, and above all should *learn to know when* they *don't know*.

LOUISA HALL.

